# Mid-Luteal Progesterone Is Inversely Associated with Premenstrual Food Cravings

**DOI:** 10.3390/nu15051097

**Published:** 2023-02-22

**Authors:** Ajna Hamidovic, Fatimata Soumare, Aamina Naveed, John Davis

**Affiliations:** 1 Department of Pharmacy, University of Illinois at Chicago, 833 S. Wood St., Chicago, IL 60612, USA; 2 Department of Psychiatry, University of Illinois at Chicago, 1601 W. Taylor St., Chicago, IL 60612, USA

**Keywords:** premenstrual, food, cravings, estradiol, progesterone

## Abstract

It is not clear whether progesterone and estradiol associate with premenstrual food cravings, which significantly contribute to cardiometabolic adverse effects associated with obesity. We sought to investigate this question in the present study based on the prior literature showing a protective effect of progesterone on drug craving and extensive neurobiological overlaps between food and drug cravings. We enrolled 37 non-illicit drug- or medication-using women in the study to provide daily ratings of premenstrual food cravings and other symptoms across two-three menstrual cycles, based on which we classified them as premenstrual dysphoric disorder (PMDD) or control participants. In addition, the participants provided blood samples at eight clinic visits across the menstrual cycle. We aligned their mid-luteal progesterone and estradiol using a validated method which relies upon the peak serum luteinizing hormone and analyzed estradiol and progesterone using ultraperformance liquid chromatography tandem mass spectrometry. Hierarchical modeling, adjusted for BMI, showed a significant inverse effect of progesterone (*p* = 0.038) but no effect of estradiol on premenstrual food cravings. The association was not unique to PMDD or control participants. Results of research to date in humans and rodents showing that progesterone has dampening effects on the salience of the reinforcer translate to premenstrual food cravings.

## 1. Introduction

Premenstrual food craving is a widespread problem in reproductive-age women [1,2,3], confirmed in both retrospective [4,5,6] and prospective [7,8,9] studies. They significantly contribute to cardiometabolic adverse effects associated with obesity. For example, self-reported premenstrual food cravings were found to be higher in the abdominally obese (defined as having waist circumference >80 cm) relative to the abdominally lean women [10], and the Study of Women’s Health Across the Nation (SWAN) identified a positive correlation between self-reported premenstrual appetite increases and high sensitivity C-reactive protein (hs-CRP) [11].

Despite the significance of premenstrual food cravings to health in reproductive age women, no study to date examined a potential effect of sex hormones (progesterone and estradiol) at their peak in the luteal (premenstrual) phase. We sought to investigate this question in the present study based on the prior literature showing a significant effect of progesterone on appetitive behaviors. Specifically, evaluations across several addictive substances show that drug craving and intake are associated with low levels of progesterone. During early abstinence, postmenopausal females with alcohol use disorder report higher alcohol cravings than premenopausal females, and in postmenopausal women, higher baseline progesterone levels correlate with lower alcohol cravings [12]. High progesterone during the mid-luteal phase of the menstrual cycle is associated with decreased stress- and drug cue-induced cocaine cravings [13]. High levels of endogenous progesterone attenuate subjective responses to cocaine cues that are preceded by a stressor (i.e., yohimbine) [14]. The administration of twice daily micronized progesterone in a double-blind, placebo-controlled manner increased smoking cessation in women during an 8 week postnatal period [15], and increases in progesterone level in non-pregnant premenopausal women is associated with increased odds for being abstinent within each week of treatment [16]. 

**Figure 1:**
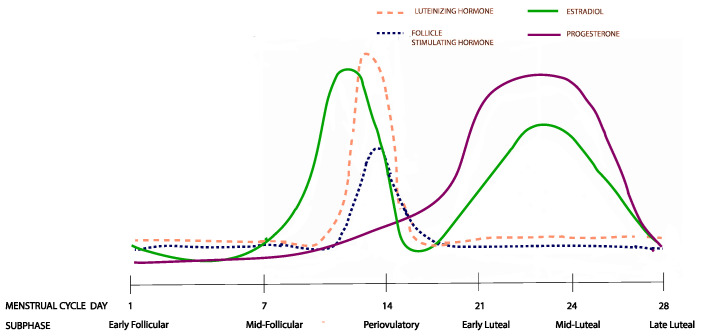
Hormonal changes across the six menstrual cycle subphases. The figure represents changes from a 28 day menstrual cycle. The cycle duration and ovulation timing, however, vary both within and between women.

The study of the menstrual cycle (Figure 1) is complicated by its natural duration variability [17], which makes the scheduling of the menstrual cycle visits difficult. Data from the study visits should correspond to the six menstrual cycle subphases that capture distinct changes in the circulating sex hormone levels: (1) early follicular, (2) mid-follicular, (3) periovulatory phase, (4) early luteal, (5) mid-luteal, and (6) late luteal [18]. An accurate and validated method for dealing with this complexity was developed in the BioCycle study [18] and it involves collecting data from eight clinic visits at the estimated menstrual cycle subphases, following which a realignment of the data is carried out based on the periovulatory LH peak.

We implemented the Biocycle protocol in the present study to accurately stage the mid-follicular and mid-luteal subphases of the menstrual cycle, and determine the effects of the sex hormones progesterone and estradiol on premenstrual food cravings. Importantly, we analyzed progesterone and estradiol using mass spectrometry, which is preferable over the immunoassay technology because it more precisely recognizes similar structures [19,20,21]. Based on the line of research that progesterone inversely associates with drug craving reviewed above and on the significant neurocircuit overlaps between drug and food cravings [22], we hypothesized that progesterone would be associated with a premenstrual increase in food cravings, and that this effect would be specific to the mid-luteal but not the mid-follicular subphase. Second, we examined whether this hypothesized association varies according to group (i.e., premenstrual dysphoric disorder vs. controls).

## 2. Materials and Methods

### 2.1. Study Design

Premenstrual Hormonal and Affective State Evaluation (PHASE) is a longitudinal study designed to increase our understanding of normal menstrual cycle physiology and its dysregulated states. PHASE is a registered clinicaltrials.gov study (NCT03862469). It enrolls women with regular menstrual cycles to chart their symptoms using the Daily Record of Severity of Problems (DRSP) [23] over two to three menstrual cycles. In the last menstrual cycle of the study, the participants complete blood and salivary sample collection visits at eight different times during the menstrual cycle, as well as psychosocial stress testing in the luteal phase and urinary LH testing. 

### 2.2. Study Sample

We recruited reproductive age women (ages of 18 and 35), with regular menstrual cycles lasting 21 to 35 days [24], from the general population using flyers, word-of-mouth referrals, and electronic media (Facebook, Instagram, and Craigslist). 

Study participants first completed an online survey, following which they were scheduled to complete an in-person screening. Before any collection of data, they signed a consent form, approved by the University of Illinois Human Research Protection Office. Study exclusion criteria were: (a) lifetime DSM-5 Axis I disorder, except anxiety and depression (based on the Structured Clinical Interview for DSM Disorders (SCID)); (b) current (i.e., within the past 12 months) DSM-5 Major Depressive Disorder or an anxiety disorder (based on SCID); (c) positive urine drug screen test; (d) breath alcohol concentration >0.00%; (e) Alcohol Use Disorders Identification Test (AUDIT) score >7; (e) self-reported smoker or carbon monoxide concentration ≥6 ppm; (f) irregular menstrual cycle; (g) current pregnancy (urine test-verified), lactation, or planning to become pregnant; (h) moderate or high suicide risk; (i) Shipley IQ (vocabulary standard score) <80; (j) prescription medications; and (k) hormonal contraception. 

### 2.3. Study Procedures

Study participants conducted urinary self-testing of the luteinizing hormone (LH) using Clearblue urine tests. They collected the first morning urine on a menstrual cycle day as specified in the Clearblue manual and continued the testing for 10–20 days, depending on whether the peak levels were reached. In order to read the results, study participants took a photo of the strip and uploaded it to the Clearblue phone app, which read the test result and determined LH as “low”, “high”, or “peak”. In the present study, this self-testing was implemented to guide the scheduling of study visits in the last menstrual cycle (last paragraph in this section), not for fertility purposes. 

On a daily basis, study participants uploaded the result screenshot of the Clearblue app into REDCap. Adherence to the daily urine LH testing procedure is critical as the capturing of the short-lived peak LH surge is based on the daily reading of LH levels [25]. In the present study, we assessed adherence to ovulation testing in real-time. On a daily basis, the study coordinator completed a checklist, ensuring compliance with the testing. In the event that a participant missed a time-point, she was contacted and asked to complete the procedure in a timely manner. 

During the last menstrual cycle while enrolled in the study, the participants were scheduled to come to the clinic for eight blood draws/saliva collection visits, as described in detail in Hamidovic et al. [26]. In the proposed study, we realigned data according to the protocol from Mumford et al. [18] and analyzed progesterone and estradiol concentrations from the mid-follicular and mid-luteal subphases in a hierarchical linear regression model, as described in detail in Section 2.6 (the Data Analysis section). 

### 2.4. Diagnosis

The Diagnostic and Statistical Manual of Mental Disorders 5 (DSM-5) specifies the possible symptoms of PMDD as: (1) affective lability (mood swings); (2) irritability or anger; (3) depressed mood; (4) anxiety or tension; (5) decreased interest in usual activities; (6) difficulty concentrating; (7) a sense of being overwhelmed or out of control; (8) change in appetite, overeating, or specific food cravings; (9) hypersomnia or insomnia; (10) fatigue; and (11) one physical symptom (for example, breast tenderness). PMDD diagnosis requires the presence of at least one affective symptom (symptoms 1–4) to reach the total of 5 required symptoms, which must be confirmed in a prospective manner for at least 2 menstrual cycles. In addition, the symptoms must be associated with clinically significant distress or interference with work, school, usual social activities, or relationships with others.

In accordance with DSM-5 criteria, PMDD diagnosis in the proposed study was assessed prospectively by evaluating the participants’ daily symptom ratings using the DRSP scale [23] during two to three menstrual cycles. PMDD diagnosis was defined as a 30% or greater increase in 5 or more symptoms, one of which had to be affective, between the luteal (day −7 to −1) and follicular (day 6 to 12) days relative to the range of the scale of each individual participant across the entire menstrual cycle [27]. They also had to have at least a 30% or greater increase in interference with daily activities due to these symptoms. PMDD participants defined using these criteria were found to have an over-expression of ESC/E(Z) complex genes [27], blunted endoplasmic reticulum stress response [28], and symptom induction upon exogenous sex hormone administration [29], which is seemingly related to specific gene induction and includes *Mtf2*—a core gene of the ESC/E(Z) complex [30]. 

### 2.5. Study Measures

#### Food Cravings 

Food Cravings. The Daily Record of Severity of Problems (DRSP) [23] is a validated questionnaire, which measures 24 symptoms of PMDD. The symptoms are rated on a scale of 1 (not at all) to 6 (extreme). The present analysis evaluated ratings of food cravings (“Had cravings for specific foods”). Once enrolled, study participants notified the research coordinator when their next menstrual cycle started. Upon notification, the research coordinator set up the DRSP surveys to be sent out daily. Study participants received a new survey link every day and were asked to complete it between 7 PM and midnight. They were also asked to use the day’s link and not any previous links to minimize retrospective reporting. In case a participant missed more than two DRSP entries in a row, or four or more in a month, the research coordinator contacted the participant to complete the survey daily. 

Estradiol and Progesterone. Estradiol and progesterone analyses were performed by the Mass Spectrometry Core in the Research Resources Center at the University of Illinois at Chicago. Analyses were conducted using an AB SCIEX 6500 QTRAP mass spectrometer coupled with Agilent 1290 UPLC system. All samples were eluted from an Agilent Poroshell 120 EC-C18 2.7 µm column (2.1 × 100 mm^2^) with a flow rate of 200 µL/min. The column compartment was kept at 50 °C. The gradient began with a 95% mobile phase A (0.1% formic acid in H2O) for 2 min and was followed by a linear gradient increase in the mobile phase B (0.1% formic acid in MeOH) from 5% to 80% in 2 min and 80% to 90% over 2 min and kept at 90% mobile phase B for 7 min, then re-equilibrated back to the initial condition (95% A) for 3 min, resulting in a total separation time of 16 min. Mass spectrometry experiments were performed via MRM scan using electrospray ionization in positive ion mode with an ESI spray voltage of 4.5 kV and source temperature of 500 °C. 

The limit of detection of estradiol and progesterone ranged from 0.05–0.25 pg and 0.5–1.5 pg, respectively. The limit of quantification of estradiol and progesterone ranged from 0.2–0.5 pg and 1.5–2.5 pg, respectively. Each calibration standard’s accuracy was within the acceptable range of 15%. The recovery of estradiol and progesterone were assessed for quality control samples at 3 levels (low, mid, and high) during the initial method development. For estradiol, recovery of 1 pg, 4 pg, and 8 pg was 99.3%, 98.6%, and 99.8%, respectively. For progesterone, the recovery of 7.5 pg, 20 pg, and 40 pg was 93.5%, 110.1%, and 95.8%, respectively. 

Luteinizing Hormone. Serum luteinizing hormone analyses were performed by ARUP Laboratories, as described in Hamidovic et al. [26].

The Beck Depression Inventory (BDI). The BDI [31] is a 21-item, self-report rating inventory that measures the characteristic attitudes and symptoms of depression. Internal consistency for the BDI ranges from 0.73 to 0.92 with a mean of 0.86. [32]. The BDI demonstrates high internal consistency, with alpha coefficients of 0.86 and 0.81 for psychiatric and non-psychiatric populations, respectively [33]. Study participants completed the BDI at screening, and the purpose was to ensure that our procedure for screening out participants with current MDD was efficient. 

### 2.6. Data Analysis

All analyses were performed in R software (version 4.0.2) [34]. To derive one summary score of premenstrual food cravings, we calculated the degree to which the DRSP food craving symptom (Section 2.5) demonstrated an elevation in the pre-menstruum (days −6 to −1) relative to post-menstruum (days +5 to +10). The average post-menstrual score was subtracted from the average pre-menstrual score and divided by participant-specific variance for the food cravings symptom across all days of the menstrual cycle. This essentially yielded an “effect size” for each woman for food cravings [7]. The effect size reflects the % increase from the postmenstrual to the premenstrual period. For example, an effect size of 0.5 indicates that there is a 50% increase in food cravings from postmenstrual cycle days +5 to +10 to the premenstrual days −6 to −1.

We first realigned the study data to ensure that all women were reclassified to the same subphase. Since the study visits occurred at predetermined timepoints, once serum LH surge was determined post hoc, the visits were realigned, and the data were standardized according to the algorithm described in detail in Mumford et al. [18], which had been completed by our investigative team previously and is described in Hamidovic et al. [26]. We analyzed the mid-luteal subphase progesterone and estradiol concentrations from the realigned dataset, which reflects the peak luteal phase progesterone and estradiol, and the mid-follicular concentrations of these sex hormones, as this subphase approximates the time of menstrual bleeding cessation. We only analyzed participants with an ovulatory menstrual cycle, defined as luteal phase progesterone ≥5 ng/mL. 

Next, we assessed the distribution of all study variables using the “shapiro.test” function. The tests of normality indicated that the distributions of mid-follicular progesterone and mid-follicular estradiol were not normal. Hence, these values were log-transformed, which normalized the data, as well as all the remaining variables. Next, we completed the hierarchical linear regression analysis. As our hypothesis was that mid-luteal progesterone would be inversely associated with premenstrual food cravings, we entered mid-luteal progesterone as step 1. We next entered mid-follicular progesterone in step 2, followed by mid-follicular estradiol and mid-luteal estradiol in step 3. Our goal was to first fit ovarian hormones, thereby characterizing endocrine menstrual cycle events (as related to progesterone and estradiol) and further build on that physiological milieu. We corrected the model for BMI (coded as underweight/normal weight and overweight/obese) in step 4 and entered the interaction between mid-luteal progesterone and diagnosis (PMDD vs. control) in step 5. Last, we visually inspected the distribution of model residuals of all the 5 steps using qq plots, and we formally tested their distribution using the “shapiro.test” function in R. 

## 3. Results

### 3.1. Study Participants

Thirty-seven women completed the study, of whom five did not have an ovulatory cycle. As our analytical approach controlled for ovulation, we completed the analysis on the remaining 32 women. Table 1 lists their demographic, anthropomorphic, and psychological characteristics. The PMDD (*n* = 13) and control (*n* = 19) participants did not differ on any characteristics listed in Table 1.

The short-lived peak LH was captured in 26 out of the 32 (~81%) participants. This rate is similar to the finding by Mumford et al. [18], which validated the study methodology we implemented in the present study, as well as in our previous publication [26]. The mean and standard deviation periovulatory serum luteinizing hormone values in the 25 participants were 34.55 (14.09) IU/L.

### 3.2. Relationship between Sex Hormones and Food Cravings

**Figure 2:**
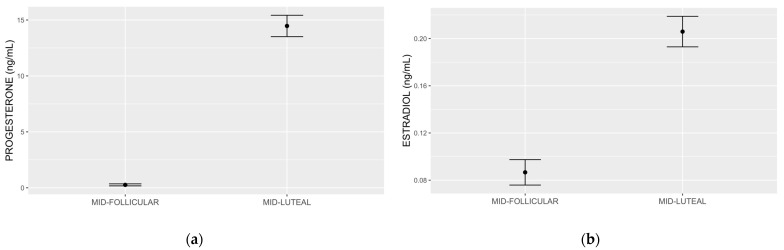
Progesterone (**a**) and estradiol (**b**) measured by ultraperformance liquid chromatography tandem mass spectrometry. The mean and standard error mid follicular and mid-luteal subphase progesterone values were 0.26 (0.08) ng/mL and 14.46 (0.95) ng/mL, respectively (**a**). The mean and standard deviation early follicular and mid-luteal subphase estradiol values were 0.08 (0.01) ng/mL and 0.20 (0.01) ng/mL, respectively (**b**).

We present mid-follicular and mid-luteal progesterone in Figure 2a, estradiol in Figure 2b, and the detailed results of the hierarchical analysis in Table 2. Step 1 of the hierarchical linear regression showed that mid-luteal progesterone (β = −0.487; *p* = 0.042) accounted for 14.9% of the variation in premenstrual food cravings (multiple R^2^ = 0.1491). Adjusting the model for mid-follicular progesterone in step 2 showed that mid-follicular progesterone is marginally associated with premenstrual food cravings (β = 0.177; *p* = 0.053), while mid-luteal progesterone remained significant (β = −0.499; *p* = 0.041). The model accounted for a greater variation (multiple R^2^: 25.0%). Introducing mid-follicular and mid-luteal estradiol in step 3 improved variation by 15% (multiple R^2^ = 40.0%), with mid-luteal progesterone remaining significant (β = −0.599; *p* = 0.020) and mid-luteal estradiol reaching significance (β = 0.495; *p* = 0.038). In step 4, the addition of BMI did not increase the variation in the outcome and mid-luteal progesterone remained significant (β = −0.692; *p* = 0.038), while none of the other predictors, including mid-luteal estradiol, showed significance. In the final model, which included the interaction between mid-luteal progesterone and diagnosis, multiple R^2^ was 52.5% and mid-luteal progesterone remained significant (β = −1.253; *p* = 0.006). The diagnosis by mid-luteal progesterone was not statistically significant. In the final model, mid-luteal estradiol was also significant (β = 0.553; *p* = 0.043). Results of the Shapiro–Wilk normality tests on individual model residuals were not significant for each of the five steps, and the visual evaluations of individual model qq plots were suggestive of normal residual distributions. Supplementary Figure S1a–e shows the individual qq plots for each of the steps. 

**Figure 3:**
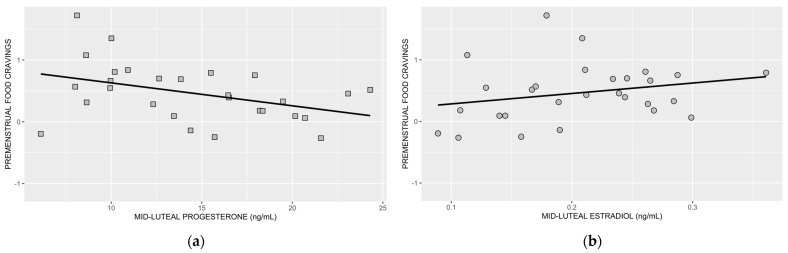
Association between premenstrual food cravings and mid-luteal progesterone (**a**) and mid-luteal estradiol (**b**). Following the inclusion of BMI, the association between mid-luteal progesterone and premenstrual food cravings remained significant (*p* = 0.038), but the association between mid-luteal estradiol and premenstrual food cravings was no longer significant (*p* = 0.080) (statistical results from step 4; R^2^ = 0.408).

In Figure 3a,b, we show associations between premenstrual food cravings and mid-luteal progesterone and mid-luteal estradiol, respectively. 

## 4. Discussion

The results of the present study demonstrate a robust inverse relationship between circulating mid-luteal progesterone and premenstrual food cravings. This relationship remained significant following the adjustment for mid-follicular progesterone, mid-follicular and mid-luteal estradiol, and BMI. The association does not appear to be unique in PMDD study participants. Although we observed a positive relationship between estradiol and premenstrual food cravings, the association did not remain significant following the adjustment for BMI.

The results of research to date in humans and rodents show that progesterone has protective effects and may dampen vulnerability to addiction [35,36]. For example, Mello [37] observed that women in the follicular phase (when progesterone is low) experience greater drug cravings, while DeVito et al. [38] demonstrated that women show diminished subjective effects (“high”, “feel good”, and “want more”) to intravenous nicotine in the luteal (when progesterone is high) relative to the follicular phase of menstrual cycle. 

Several research domains demonstrate neurobiological overlaps between drug and food cravings. Craving in the laboratory is provoked via cue presentation, which activates the prefrontal cortical regions to drive the process through functional connections with the striatum [39]. The activation in the prefrontal cortical regions following cue presentation correlates with cravings for drugs or food [39,40]. Pharmacological treatments can target both drug and food craving. Buproprion/naltrexone combination—an FDA-approved adjunct medication to a reduced-calorie diet and increased physical activity for chronic weight management in obese adults—produces a significant reduction in food cravings [41,42]. Buproprion, an FDA-approved medication for smoking cessation, reduces nicotine cravings [43,44], while naltrexone, an FDA-approved medication for alcohol use disorder and opioid use disorder, reduces alcohol [45,46] and opioid [47] cravings. Non-pharmacological interventions—repetitive transcranial magnetic stimulation (rTMS) and transcranial direct current stimulation (tDCS)—effectively reduce food and drug cravings [48]. Combined, these studies unequivocally point to a shared neurocircuitry between food and drug cravings, reflected in the results of both experimental and clinical research. Hence, our results—showing progesterone dampening effects on food cravings—are in line with the literature on addiction, demonstrating the same effect across several addictive drugs [12,13,14,15,16].

Cellular and anatomic brain circuits underlying the drive for food are highly controlled and very complex. Reinforcing drugs—as well as palatable foods—may overwhelm these control mechanisms, leading to the abnormally enhanced salience of the reinforcer. The brain systems driving the motivation to promote excessive feeding and uncontrollable drug intake have significant overlaps, although the regulation of food intake is much more complex [49]. While drug intake is predominantly mediated by the rewarding effects of drugs, food intake is controlled not just by its rewarding effects, but also by multiple peripheral and central homeostatic factors [49].

The identified inverse relationship between progesterone and food cravings likely represents a hedonic, not a homeostatic, process, though this hypothesis needs to be tested in the future. Changing sex hormones in the luteal phase are associated with decreased levels of amino acids and lipid species, presenting a physiological milieu of a heightened energy requirement [50,51,52,53,54,55]. Progesterone upregulates cell cycle and growth, resulting in protein biosynthesis for endometrial thickening [56]. If the relationship between progesterone and food cravings reflected this particular homeostatic process, then it would have been positive, as higher progesterone would have resulted in higher food cravings due to a small but significant positive effect of the luteal phase on resting energy expenditure [57]. 

Whether the heightened food cravings associated with low progesterone identified here actually translate to an increased food intake needs to be investigated in a rigorous study which controls for ovulation, periovulatory subphase (determined by serum luteinizing hormone), a precise subphase alignment, and measurement of sex hormones via mass spectrometry. Roney and Simmons [58] examined within-cycle shifts in total food intake and sex hormones (estradiol and progesterone), showing that progesterone positively and estradiol negatively predicted food intake, especially during the periovulatory subphase when there is a significant drop in food intake. Study participants collected salivary samples for a further concentration analysis of sex hormones using the immunoassay technology. However, relative to the mass spectrometry gold standard for the measurement of progesterone, the immunoassay analysis of salivary progesterone may produce skewed results. For example, Ney and colleagues [59] analyzed salivary samples for progesterone using mass spectrometry and immunoassay, showing that progesterone was highly variable and overall significantly higher when analyzed using immunoassay compared to mass spectrometry, mostly due to its cross reactivity with 17α-hydroxyprogesterone (17α-OHP). The authors concluded that research using salivary progesterone immunoassay techniques should be interpreted cautiously due to a high variability of results from the immunoassay measurement. Mass spectrometry methods are starting to become the reference method for the analysis of both sulfated and non-sulfated steroids in clinical laboratories and research studies, as they provide high accuracy [60]. Though the cost of mass spectrometry techniques is currently high, this is expected to decrease over time. Hence, the relationship between sex hormones and food intake, in particular in the luteal phase of the menstrual cycle, is still an inconclusive area which needs to be further explored.

We suspect that the inverse effect of progesterone on food craving had not been captured until now due to the immunoassay measurement of progesterone in studies to date, and also because the mid-luteal subphase may not have been properly aligned. As endogenous progesterone kinetics follow an inverted u trajectory across the luteal phase, if the subphases are not perfectly aligned, women in the study may be misclassified for being in the low or high progesterone group. We further suspect that the inverse association identified in the present study reflects the dampening effect of progesterone on rewarding processes. Indeed, the luteal phase of the menstrual cycle is marked by an increase in palatable (hedonic and rewarding) food intake, as shown in the study by Gorczyca et al. [61], which identified increased cravings for chocolate, sweets, salty flavor, and general food cravings. With respect to the mediation of reward circuits, the attenuating effects of progesterone involve endocannabinoid, γ-aminobutyric acid, dopamine, and glutamate transmission in the medial prefrontal cortex and striatum [62]. The neurobiological bases for the identified association in the present study warrants a further investigation.

Our study had strengths and limitations. Ideally, the present study should be replicated in a larger sample of study participants. However, any sample size needs to be weighed against sources of bias. The present study recruited a sample of women with no illicit drug- or prescription drug-use, who did not smoke or consume heavy amounts of alcohol, all of which may distort circulating sex hormone levels, thereby removing a number of potential confounding effects. We excluded individuals with positive drug urine screenings, heavy alcohol drinking, and current smoking to better define effect size estimates (i.e., removing significant sources of bias). Similarly, we excluded women with current major depressive disorder, though that may have limited the generalizability of the study findings given the significant comorbidity between the two disorders [63,64]. Moreover, although the Daily Record of Severity of Problems (DRSP) is a validated instrument for the collection of premenstrual symptomatology, food craving is only one item; hence, the measurement of craving ideally should be examined using more powerful methodologies, such as laboratory, cue-induced cravings. The study’s strengths are a prospective measure of premenstrual food cravings, the adjustment of premenstrual food craving relative to the scores from the follicular phase (and variance of all ratings across the entire menstrual cycle), the accurate staging of the mid-luteal subphase, the adjustment for BMI, and the implementation of ultraperformance liquid chromatography tandem mass spectrometry, which demonstrates high steroidal specificity [65].

In summary, the present study shows the protective effects of progesterone on premenstrual food cravings. The significance and the direction of this novel finding is in line with the existing literature demonstrating an inverse relationship between progesterone and drug cravings. Future studies should evaluate the neurobiological mechanisms of this finding.

## Figures and Tables

**Table 1:** Demographic and anthropomorphic characteristics of study participants with an ovulatory cycle (*n* = 32).

Category	Mean (SD) or N
AGE	
	26.53 (4.93)
RACE	
White	12
African American	7
American Indian/Alaska Native	1
Asian	7
Native Hawaiian or Other Pacific Islander	0
More than 1 race	1
Unknown/Do not want to specify	4
ETHNICITY	
Hispanic	10
Non-Hispanic	20
Unknown/Do not want to specify	2
STUDENT STATUS	
Yes	17
No	15
MARITAL STATUS	
Single/Never married	28
Married	3
Divorced	1
INCOME	
Less than USD 20,000	5
USD 20,000–USD 34,999	15
USD 35,000–USD 49,999	4
USD 50,000–USD 74,999	5
USD 75,000 or more	3
MENARCHE AGE	
	11.86 (1.43)
BMI *	
	25.48 (4.71)
BDI **	
	2.65 (3.00)

* BMI = Body Mass Index. ** BDI = Beck’s Depression Inventory.

**Table 2:** Results of hierarchical regression with mid-luteal and mid-follicular sex hormones, BMI, and mid-luteal progesterone by diagnosis as predictors of premenstrual food cravings.

	Estimate	Std. Error	*t* Value	*p* Value
STEP 1				
Mid-luteal progesterone	−0.487	0.228	−2.134	0.042 *
STEP 2				
Mid-luteal progesterone	−0.499	0.230	−2.165	0.041 *
Mid-follicular progesterone	0.177	0.087	2.048	0.053
STEP 3				
Mid-luteal progesterone	−0.599	0.237	−2.524	0.020 *
Mid-follicular progesterone	0.144	0.082	1.741	0.097
Mid-luteal estradiol	0.495	0.223	2.214	0.038 *
Mid-follicular estradiol	0.033	0.138	0.236	0.815
STEP 4				
Mid-luteal progesterone	−0.692	0.311	−2.226	0.038 *
Mid-follicular progesterone	0.167	0.097	1.717	0.102
Mid-luteal estradiol	0.452	0.245	1.846	0.080
Mid-follicular estradiol	0.052	0.147	0.352	0.729
BMI	−0.105	0.220	−0.478	0.638
STEP 5				
Mid-luteal progesterone	−1.253	0.407	−3.077	0.006 **
Mid-follicular progesterone	0.150	0.093	1.614	0.125
Mid-luteal estradiol	0.553	0.254	2.176	0.043 *
Mid-follicular estradiol	−0.046	0.148	−0.311	0.760
BMI	−0.129	0.214	−0.603	0.554
Mid-luteal progesterone * diagnosis	0.967	0.476	2.031	0.058

* *p* ≤ 0.05; ** *p* ≤ 0.01.

## Data Availability

The data presented in this study are available on request from the corresponding author. The data are not publicly available to preserve scientific integrity of research methodology.

## Supplementary Materials

The following supporting information can be downloaded at: https://www.mdpi.com/article/10.3390/nu15051097/s1, Figure S1a–e: QQ plots of the five-step hierarchical linear regression modeling.
