# Supplementary material for: Mid-Luteal Progesterone Is Inversely Associated with Premenstrual Food Cravings
Source: Nutrients. 2023 Feb 22;15(5):1097. doi: 10.3390/nu15051097 (PMC10005553; doi:10.3390/nu15051097)

Supplementary Figures S1 a-e. QQ plots of the five-step hierarchical linear regression modeling

Step 1 (Supplementary Figure S1 a)

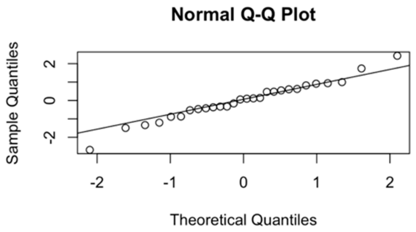

Step 2 (Supplementary Figure S1 b)

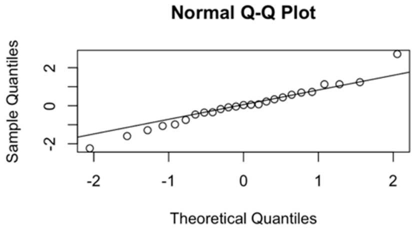

Step 3 (Supplementary Figure S1 c)

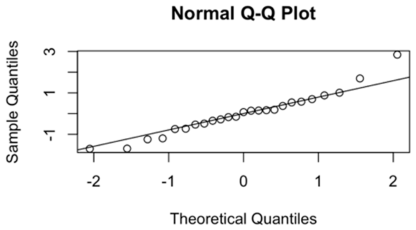

Step 4 (Supplementary Figure S1 d)

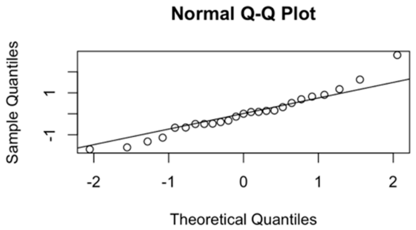

Step 5 (Supplementary Figure S1 e)

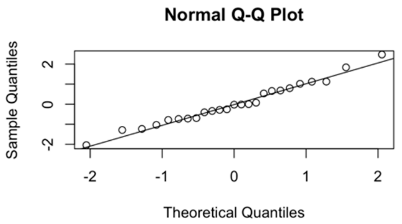

Supplement: Supplementary file 1 [file nutrients-15-01097-s001.zip › nutrients-2213850-supplementary.pdf]
